# “I kind of gave up on it after a while, became too hard, closed my eyes, didn't want to know about it”—adults with type 1 diabetes mellitus describe defeat in the context of low social support

**DOI:** 10.1111/hex.12850

**Published:** 2018-11-22

**Authors:** Kathleen Hill, Paul Ward, Jonathan Gleadle

**Affiliations:** ^1^ Discipline of Public Health Flinders University Adelaide South Australia Australia; ^2^ School of Nursing and Midwifery University of South Australia Adelaide South Australia Australia; ^3^ College of Medicine and Public Health Flinders University Adelaide South Australia Australia

**Keywords:** family, health service, social support, type 1 diabetes

## Abstract

**Background:**

Type 1 diabetes mellitus (T1DM) is a lifelong condition that requires diligent self‐management to avoid complications. Living with T1DM is a considerable challenge and the inability to follow a prescribed regimen is often termed non‐compliance. However, this fails to acknowledge that for some people the barriers to glycaemic control may be insurmountable.

**Objective:**

This qualitative study explores the structural determinants, social context and lived experience of T1DM with 17 adults to understand influences on patterns of self‐care, engagement with and trust in health‐care services, and health outcomes.

**Results:**

Their stories tell us that strong social support is vital to disease adaptation and ongoing management. When social support is absent, the story is one of struggling with intensive diabetes management alone and difficulty controlling blood glucose levels. When confronted with suboptimal glycaemic control, participants isolated from social support developed combative relationships with health‐care providers and disengaged from health care. Their subsequent slide to chronic comorbid illness is steep and this study reveals the heartache and loss experienced when difficult life circumstances and low levels of social support have led to irreparable kidney damage.

**Conclusion:**

Patterns of poor glycaemic control viewed in the health‐care encounter without an understanding of the context or life circumstances in which they are occurring can lead to an inability to engage with health‐care services. Disengagement from services and the absence of specialist care further isolates people, leaving them managing their diabetes alone with limited success.

## 1 INTRODUCTION

Type 1 diabetes mellitus (T1DM) is a serious chronic disease characterized by the inability of the pancreas to secrete insulin and onset is rapid occurring over a number of weeks. Individuals with T1DM are subsequently dependent on insulin injections for the rest of their lives and require an intensive daily programme of dietary management, blood glucose level monitoring and frequently adjusted doses of insulin. The potential health complications of living with T1DM can be delayed through good concordance with therapeutic regimens but self‐management of T1DM over a lifetime is an enormous challenge even in favourable social and environmental circumstances.^1^ Social factors that affect health and well‐being, described as social determinants of health, are shaped in early childhood environments and set individuals onto life course trajectories that cannot be changed by individual choice alone.^2^ Social determinants of health involve a large number of social factors operating in the same direction^3^ creating either a negative or a positive social environment. The importance of feeling secure, safe and loved in childhood has strong links to feelings of self‐worth, self‐efficacy and subsequent adult well‐being.^4^, ^5^ In addition, our worldview is developed in childhood and a positive early childhood environment leads to a worldview that is characterized by trust in others and the ability to form trusting and intimate adult relationships^6^ that foster high levels of social support. Feelings of trust are integral not only to the ability to form close attachment relationships in adulthood, but also to the ability to develop concordant relationships with health‐care providers, which is vitally important in relation to the management of lifelong chronic disease.^7^, ^8^

Reduced social support in the context of low socioeconomic environments is a multifactorial mechanism that can underlie poor outcomes in health^2^ because, even in the context of low socioeconomic status (SES), feeling cared for, valued, belonging and under mutual obligation has a moderating effect on stressful environments.^9^ The perception of trust can also be diminished when the person affected by chronic disease is burdened by low socioeconomic status and diminished social support,^10^ because these social factors can reduce health literacy and participatory interactions with health‐care providers, and hence reduce the individuals capacity for health. The focus of this study was to explore social factors that can affect self‐management in T1DM and the mechanisms through which this occurs. This study also describes what is lost when unsupportive social environments lead to the development of health complications at a comparatively young age. The absence of qualitative research with adults who can describe the lived experience of T1DM, and give insights into how health‐care services can better meet the needs of the people most vulnerable to dropping out of care, has been one of the most pressing gaps in the literature.^11^

## 2 METHODS

Qualitative research is the exploration of a phenomenon achieved through the deep probing of an individual's perceptions of events, which encourages participants to define their experiences in their own words and on their own terms. This study sought elucidates the psychosocial enablers and barriers to optimum glycaemic control. The conceptual framework of critical social theory guided the enquiry, which seeks explanation for consequences that are fashioned by social forces. This qualitative enquiry is an exploration of these forces in relation to the self‐management of T1DM, and “steps back” from individualism and reflect on the conditions under which people act. The enquiry is centred on social determinants of health which are measured as concepts in the qualitative data. The primary focus is on describing the increased risk or susceptibility to adverse health outcomes related to lack of social and environmental resources and the supportive elements of a favourable environment in relation to disease outcomes. The operationalized measures include employment, income, education, housing and social support including social cohesion, social inclusion and social empowerment.^12^, ^13^ Other important factors explored are access to and quality of health care including ease of access, retention to care, engagement with services, patterns of attendance, degree of interpersonal and institutional trust.^8^

### 2.1 Ethical considerations

The researchers obtained ethical approval to conduct this research and all participants in the study gave informed written consent.

### 2.2 Participants

Participants for this study were purposively sought from an endocrinology and renal department through the placement of posters advertising the study and through clinician support in promoting the study to people attending the clinic. People who expressed an interest in being involved were provided with the participant information and consent form (PICF) to consider and contacted by the researcher one week later by phone. Twenty‐two people received the PICF and 17 opted to be involved. This heterogeneous group of adult participants comprised of 10 males and seven females with an age range of 23‐62. In addition, the group had a wide range of disease duration from three months to 53 years and were able to share a variety of experiences from recent diagnosis to the challenge of living with T1DM over many years.

### 2.3 Semi‐structured interviews

All interviews were audio recorded, occurred in the health service setting in a private room, were one‐hour average duration and transcribed verbatim.

### 2.4 Data analysis

The interview data were read repeatedly and coded for common expressions and experiences. The codes were then collated and operationalized into common themes. Data were thematically analysed with the support of the software program NVivo (QSR Nvivo10 2014, QSR International, Melbourne, Australia). The analysis occurred in tandem with recruitment to the study in order to collect the most relevant data from the interviews as the emerging themes came to light. The thematic analysis trended towards data saturation with regard to managing T1DM and engagement with health services by interview ten; however, the mechanisms underlying disengagement from health‐care services with resultant severe health complications were difficult to elucidate and data collection continued until this phenomenon could be more fully understood.

## 3 RESULTS

The results of this study describe the important role of support for adults with T1DM. Support is multifactorial and lies in family, close relationships, but also in the relationships that the person with T1DM is able (or not) to develop with their health‐care providers (HCP) which is an integral part of managing a complex health disorder. This research illuminates some of the reasons people with T1DM stop coming to clinic and as such the results are presented to demonstrate the disruptive nature of the diagnosis, family support, social support and the multiplicity of disengagement from health services with resulting catastrophic disease progression.

### 3.1 The diagnosis experience is life changing

The participants describe the diagnosis of T1DM as abrupt, confusing and life changing. There is little opportunity to adjust and people carry this experienced with them throughout their lives marking a time point in which there was significant change. Participants with childhood onset had imperfect recollections of the diagnosis experience but remembered clearly being in hospital as a child and feeling isolated from family;

P6 “I'd never really been in hospital for anything major like that before and it was pretty much ‘all right, here's your insulin’ My aunty gave me the injection and pretty much ‘we'll talk to you in the morning’ so I just laid there, just sort of not really getting what's going ‐ you know, understanding it but not understanding it” (teen onset)

When onset was in adulthood, the day of diagnosis and commencement of therapy was recalled by participants as a surreal experience. The blunt nature of this experience was recalled as a terminal event, with life changing dramatically from that moment on. Participants described receiving education around the time of diagnosis whilst also experiencing a sense of transformation into a new life with diabetes leaving them overwhelmed;

P7 “The first educator said it's not really a life changing disease and a week later I was completely – I completely disagree with *” (adult onset)

*gender substitute

P9 “When I left hospital I didn't really know what I was doing. They gave me some needles and said ‘you need to inject. There you go’. I was left pretty quickly ‘here's a bag. Here's everything in it. Test your blood, take six units with every meal, call us with your readings’. That part was very quick and out the door” (adult onset).

Participants were also able to describe their parents’ reactions to the diagnosis, which tended to involve a sense of grief and loss irrespective of the age of onset;

P9 “My mum was heartbroken. My mum was really shocked that I got it. I think she was more upset and worried about it than I was. She just kept crying and saying that if she could take it away she'd have it so I wouldn't have to have it”(adult onset)

P5 “Mum cried a lot and I had to deal with her being really upset” (childhood onset)

The abrupt and life changing diagnosis of T1DM can be traumatic and highlights the importance of family support during this time.

### 3.2 The role of family and social support

Fortunately, clinical care and education in T1DM is evolving, never‐ending and with constant repetition, practice and support leads to eventual mastery in diabetes management. Mastery is described by the participants in this research as finally gaining a complex understanding of their *individual* glycaemic responses to food and insulin administration, which allowed in most cases the ability to predict what blood glucose levels were likely to be and how the day looked for them in terms of management;

P4 “The diabetes is – you have to be very, very conscious of it. It's a sneaky disease” (childhood onset)

This however becomes an entirely different scenario when faced by a person with low levels of social support and low levels of self‐esteem. The idea that early childhood environment is a social determinant of health was explored with all participants being asked to describe with whom they lived when growing up and to discuss in detail their relationships with their parent(s). Seven participants came from a single‐parent family; however, growing up in single‐parent families did not always result in poorer outcomes in T1DM. It appears that maternal relationship was the critical factor in supporting participants, whether in a one or two parent family;

P9 “(I) Sat and spoke to my mum and she was like ‘you're trying to be the perfect diabetic; there isn't one” (adult onset)

P16 “I don't reckon she pushed me (to self‐manage). I reckon it was when I was ready to do it. Mum wasn't like that; she'd just go on and on” (childhood onset)

And when the maternal relationship was not strong;

P1 “because I think she didn't understand it she was a bit scared about it because she had no idea how to deal with it or anything like that and she didn't know what it meant, like in the way of what I had to do to look after myself after that, so she was always – I think it's because of the way she was brought up as well. She sort of thought she – without realising it, she pushed me away more than helped me accept it and stuff”.

A strong theme in the interview data was the relationship between low maternal attachment and an inability in adulthood to form close attachment relationships that could be a source of support in diabetes management. Participants who recalled adverse early childhood environments with low maternal attachment that resulted in leaving the family home in early teen years reported subsequent tumultuous adult relationships and either not being in a relationship at the time of the study or a history of low‐quality relationships in adulthood;

P 10 “I've had two husbands and I've had three long term relationships, I've always felt that I choose the wrong people because anybody that shows me a bit of love and affection I will just – I latch on because that's what I'm missing and that's what I'm looking for”(teen onset)

For participants, adverse early childhood environments with low parental attachment affected their life trajectory in a cyclical way by inhibiting the ability to bond closely in adulthood, which led to ongoing low levels of social support. In direct contrast, participants with positive parental attachment also reported strong attachment relationships in adulthood and the presence of a support person who helped with diabetes management. Participants who described feeling that their diabetes was under good glycaemic control also recounted positive childhood environments and spoke of their support person in adulthood being closely involved in the day‐to‐day management of diabetes and navigation of the health‐care system. This increased engagement with health‐care professionals and promoted an active interaction from which there were significant gains;

P 12 “I just relate it as to like a formula one driver. So I'm like the driver who's doing everything but they're (partner and endocrinology team) the pit crew and they're everyone who does all the maintenance side of things behind the scenes, so you couldn't do it by yourself; you couldn't do it” (adult onset)

Whilst T1DM is often referred to as a self‐managed condition, these findings suggest that social support is key to successful management. A central component of the day‐to‐day treatment is the presence of a supportive person who understands diabetes and what needs to be done. For participants, this strong social support took the form of practical support such as medication management, dietary support and liaising with HCP but it also involved social support, often described simply as having someone to talk to;

P 13 “* and I talk about it a lot. I think I used to probably complain a lot about it and I didn't want * to do anything about it, it was just like * was a backboard for me.” “Sometimes you've just got to let it out and tell someone and I think it probably gave * more understanding of what you go through sometimes” (adult onset)

*name and gender substitute

### 3.3 The role of the health‐care provider

In direct contrast, low levels of social support were described by seven participants who had struggled with their diabetes management, had a history of poor glycaemic control and the development of complications and, without exception, the absence of specialist care. These factors appear to be interconnected, with low levels of social support leading to erratic and difficult glycaemic management, with no one to turn to for help and a sense of isolation in the health‐care encounter making it more difficult to navigate;

P10 “You're struggling with trying to cope with having the disease but you're also struggling with trying to cope with other people's reactions towards you and you're struggling to sort of cope with how do I fit” (teen onset)

This is particularly well expressed in describing the transition from paediatric services into adult services;

P14 “I think that's one of the main things and I think that's why I didn't like going, as I said because I just felt like they didn't give a shit ‐ didn't care much. As I said I don't think they cared for the patients much. I just think you were a number, you know, patient number 20, patient number 21. They just wanted to get through it and go home at the end of the day; that's what I think it was like in the adult place” (childhood onset)

Managing diabetes alone, with little or no social support and no medical or allied health support, is an insurmountable task which results in suboptimal glycaemic control for long periods of time and the silent undetected advancement of severe complications.

Participants who described their background as low socioeconomic status were able to explain in much greater detail the mediators for poorer outcomes. Low levels of social support, low health literacy and low self‐esteem manifested as an invidious distrust of the endocrinologist and an inability to engage with care;

P17 “That was another part of the problem, I found the* (endocrinologist) a bit off putting, * was a bit up *self, I used to walk into * office, a room like this and there would be this big carved wooden table and this big chair and * was in (place) and all that stuff, kind of rubbed me the wrong way, * was a bit elitist you know, and I didn't feel comfortable, I don't know why, looking back” (adult onset).

*gender substitute

This is an important social factor that leads to withdrawal from care. A theme that emerged from all participants in describing their health‐care encounter was the need to be recognized as someone with the capacity to contribute to the discussion in a meaningful way. This ensures that participation in the encounter is active and not passive. Health literacy as a concept in health care is most often thought of as ability in literacy and numeracy but in fact it encompasses much more. A vital aspect of health literacy is the ability to engage in a meaningful way with health‐care practitioners developing a concordant relationship that enables joint decision making and common goals. Participants emphasized the importance of this, describing a keen need for health‐care practitioners to acknowledge their expertise as the person with the disease and to consult them on changes that needed to be made to the treatment regimen. Participants who were having problems managing their diabetes needed help and did not want to be criticized or blamed, but their inability to articulate this resulted in a passive health‐care encounter from which they received very little;

P 1 “I think if we focused more on the things that I can do and the benefits that it would give me if we did that, then I probably would have accepted it better” (teen onset)

Passive health‐care encounters also occurred when the person with diabetes felt they were being blamed for their poor glycaemic control. They did not view this as a personal choice but felt their HCP did, and they became passive and disengaged from the discussion when that occurred;

P14 “It was basically ‘your thing's high (HbA1c). We've told you what to do but you're not listening so why waste our time?’ That's what it felt like” (childhood onset)

P11 “Yeah and when you've got it stable and settling it down you feel so much better and you can talk about it (with the HCP), you can – yeah” (childhood onset)

Participants that had been unable to establish a strong ongoing relationship with a health‐care practitioner described care that consisted of having prescriptions filled with no surveillance for kidney disease until the abrupt and sudden diagnosis that kidney disease was well advanced at a comparatively young age:

P15 “Went into the GP for some antibiotics for a cough that wouldn't go away and they said ‘oh we'll just take a blood test, just to check that everything's all right’ and that's when the kidney function came back at 19 percent, and that was the first test that was done with a kidney function test on it, so I went from thinking I was healthy to end stage kidney failure. If it had been tested six months, a year, two years beforehand it probably would have shown up but it just wasn't” (child onset).

Disengagement from health‐care services was described as occurring in the setting of difficulty managing the condition very early in the diagnosis, or in the immediate period following transition to adult services and to an intensive regimen. Poor glycaemic control and subsequent combative relationships with health‐care providers preceded all cases of disengagement. The downward cycle had multiple elements that started with low levels of social support, an inability to master the treatment regimen and achieve glycaemic control and an inability to engage with health‐care professionals. These participants described their HCP whom they thought “talked down to them” and made them feel as if they were wilfully disregarding the advice given and that poor glycaemic control was a personal choice. These participants acknowledged that there were periods when they took their standardized dose of insulin to stay alive, did not monitor their blood glucose levels and consequently developed a fatalistic attitude about the potential of the health complications they were now experiencing;

P17 “Kind of gave up on it after a while, became too hard, closed my eyes, didn't want to know about it” (adult onset).

The fact that scenarios like these have been described has important implications for clinical care. This is best demonstrated by participants who returned to endocrinology care after the development of kidney disease. They carefully and insightfully described their strong need to be judged not by the mistakes they made in the past but by what they needed now;

P15 “I imagine that there would have been a little bit of ‘come on, what have you done?’ as people ‐ it's just how people are but I just said ‘yeah, look, this is the current state of play. My goal is to take better care of my sugars so that I avoid any more further damage; how do I do it?’ So I've come out and just said straightaway ‘look, you specialist, me patient. Let's work together. Let's get it done”(child onset).

The catastrophic impact of severe diabetes complications on a young person in what should be the prime of life cannot be underestimated. Much is lost when an adverse childhood environment leads to low levels of social support, difficulty achieving glycaemic control and disengagement from care;

P17 “My life has been ruined by it, I'm (age), I've had my feet amputated, got kidney disease, my eyes are playing up, I can't read without my glasses, it cost me my job, it's cost me a lot” (adult onset).

P 2 “Put me on sickness benefits and put me on the dole and sit me in a room and I'll smack my head against the wall for the rest of my life” (childhood onset).

## 4 DISCUSSION

### 4.1 Biographical disruption

The diagnosis of a chronic illness can create “biographical disruption” where not only is the physical body affected but the whole life trajectory.^14^ The diagnosis of T1DM in a child appeared to induce a sense of grief for parent/s, with childhood onset participants recalling their parent/s being terribly upset, and consequently they themselves feeling guilty for upsetting the family. This parental grief with components of both fear and sadness is similar to that seen in other childhood chronic illness including asthma and arthritis.^15^ Parental support, when positive, supported this biographical disruption and the transition to self‐care occurred slowly and in a supported way that fostered a degree of safety and control. Participants with low levels of maternal attachment described a sense of confusion, isolation and helplessness when their lives were abruptly disrupted by the diagnosis of T1DM. When the diagnosis of T1DM was in adulthood, it was described as a psychologically traumatic event, creating a sense of shock and a clear divide between life as it was and life as it now will be. The abrupt and terminal nature of this event in adulthood needs both good social support and good support by health‐care professionals with frequent contact in the first few weeks to avoid a sense of isolation. The process of adaptation to the diagnosis was long and sustained and required intensive support from the endocrinology service, without which full adaptation failed to occur and living with T1DM became a constant battle to maintain blood glucose levels in a stable range. This would lead to the phenomenon known as diabetes specific distress, which relates to an anxiety or depressive response to the demands of the treatment regimen and the constant pressure that is felt. What is hoped in the diagnosis of a life changing condition is that biographical disruption becomes biographical reinvention, when people recover from the disruptive effects of the diagnosis and develop new ways to live with the condition.^16^ Vital components of this process however are good social support and quality HCP support.

### 4.2 Family and social support

This study has demonstrated that entering adulthood with a strong sense of self‐determination fostered by a positive early childhood environment and high levels of social support led to mastery in T1DM. Social conditions are “fundamental causes” that influence how people are exposed to individually based risk.^17^ Maternal attachment is a vital element in the development of self‐determination. Participants in this research with a history of strong maternal support described how they adapted more readily to T1DM diagnosis and treatment regimens and reached autonomy in diabetes care in a supportive environment. Family breakdown in relation to parental separation has been described previously as a factor in poor glycaemic control,^18^, ^19^ but participants in this study reported this as mediated by strong parental attachment that remained intact during and after the parental split. This early childhood parental attachment appears to increase capacity in developing strong attachment relationships in adulthood subsequently resulting in an environment of good social support.

In the absence of support, individuals struggle with problematic glycaemic control, have difficulty engaging with health‐care services and express a sense that management of T1DM is too hard, giving up and having just the very basics of glycaemic care in place. Social support has components of instrumental support, companionship, emotional support, relationships and connections.^20^ This research has demonstrated that high levels of social support are also predictive of positive engagement with health‐care services and that the absence of social support leads to inability to build active partnerships with HCP to support self‐care. In diabetes care, having high resources in relation to social support leads to a higher motivation to do well and it is important therefore to consider the social context that makes choices real and available.^21^ People that manage diabetes well are advantaged in their social resources and this support enables them to succeed and to become “health capable”.^22^

### 4.3 Capability

The concept of being able to make a rational choice, which arguably underpins contemporary chronic condition self‐management, fails to take into account that “agency” refers not only to intent but also to a person's capability and it is necessary to measure both to understand unintended health consequences.^23^ This research has used a critical social theory perspective to explore participants’ individual agency and the social structures and mechanisms that shape the experience of living with T1DM, including factors that supported or hindered diabetes management, and the importance of engagement with and trust in health‐care services. People are born with different capabilities and society often blocks certain groups from reaching these by social exclusion and the top‐down approaches taken in contemporary health‐care settings. Top‐down approaches are patriarchal and disempowering^24^ and do not allow people to reach their full potential. This will only be achieved by HCP adopting an assets versus deficits model of care^25^ that works with people to identify their strengths to realize their potential.

### 4.4 Redefining health‐care services

Australia has a well‐developed health‐care service that offers universal access and it is known that inequalities in diabetes outcomes can disappear after compensation with treatment and education at a tertiary care centre.^26^ However, adults who feel poorly equipped to manage T1DM may also experience difficulty navigating the health‐care system, and in what is described as the inverse care law^27^ those who need it most may also be less likely to receive care. Compensatory mechanisms can be put in place to address the inverse care law by providing additional services to those with the highest need. However in diabetes care, this often does not occur due to glycaemic control being viewed as a result of disposition rather than situations resulting in the phenomenon of “compensatory inversions” where additional resources are then offered to and accessed by those with the least need for them.^3^ Some of the participants in this research described feeling that health‐care services had let them down. There was a duality in their discourse between self‐blame and system‐blame, with participants who had not fared well with their diabetes management describing their struggles with glycaemic control, difficulty adapting to an intensive regimen, and feeling intimidated and under resourced to manage their encounters with HCP. This in turn led to a passive encounter in which little was gained and eventually resulted in disengagement from care with dire consequences. The health‐care encounter has a component of health literacy^28^ which is the ability to interact in that environment in a positive way by being an active participant in the decision‐making process, and taking from the encounter tools to support living with diabetes. The participants in this study at times blamed themselves for their inability to manage their diabetes, but they also gave strong accounts of where they thought the system should be blamed, for failures in offering sufficient support and in listening to their individual stories and the problems that they have. Mechanisms of adverse outcomes in diabetes care for people with adverse social environments can be found in the design and implementation of treatment regimens.^3^ In the case of T1DM, intensive diabetes management needs to be supported with intensive health service delivery. This is particularly so when considering the need for additional services to be directed to the very groups that are also the least likely to remain engaged with care. The accountability for poor outcomes, whilst most often attributed to the individual, also lies with health‐care services that allows people with T1DM to collect insulin prescriptions and consumables over many years without active endeavours to refer them to specialist care and to screen for complications. Previous qualitative research has demonstrated that the fear of being judged for poor glycaemic control results in clinic avoidance^29^ and high HbA1c levels may lead to clinic non‐attendance and the beginning of a downward spiral. Where disengagement cannot be avoided, limited services should be maintained, particularly screening for complications, and there need to be ways of welcoming people returning to care. Services can fail to meet the needs and preferences of their patients leading to high attrition rates because of the absence of joint consultations and shared decision making^30^ and this is particularly true for adults with T1DM and further compounded for those with adverse social environments.

Adults with T1DM need intensive sustained support to self‐manage this lifetime chronic illness and current service provision has several faults. Research in diabetes continues to focus on the absence of formalized psychological treatments, which whilst effective in children and adolescents are thought to have no sustained effect in adults with T1DM.^31^ The participants of this study clearly described a need for someone to talk to, without judgement, and this is not described as formalized psychology but as the need to reach out to another person to describe how they were feeling about their health. A novel concept would be to trial the introduction of components of “pastoral” care into diabetes care. Pastoral care has undergone significant reform to meet the needs of modern communities and has evolved into a fundamental component of child education to support healthy emotional, social and personal development with acknowledgement that social isolation can be similar to depression.^32^ Components of pastoral care that have been successfully incorporated into diabetes services providing multidisciplinary collaborative models of care include life coaching, group classes and customized feedback.^33^ Endocrinology services need to evolve from the traditional didactic model of care to one that has continuity in care, builds patient‐provider relationships by incorporating a detailed understanding of the complexity of an individual's social history, and understands that the fear of being judged is a critical concern for people with diabetes and a known barrier to clinic attendance.^34^

## 5 CONCLUSION

This qualitative study has explored the lived experience of T1DM and the impact of the social environment through in‐depth interviews with 17 adult participants with highly varied life circumstances and disease trajectories. The results have demonstrated that a positive early childhood environment, particularly maternal attachment, is a factor in the development of a sense of self‐determination and resilience in diabetes care. Participants with low maternal attachment tended to have low levels of social support and were under resourced for the challenges of managing a highly complex disease. HCP may view a pattern of poor glycaemic control as a rational choice when in fact it is no choice at all because the challenge has become insurmountable in the absence of strong external support. Patterns of poor glycaemic control, viewed in the health‐care encounter without an understanding of the context or life circumstances in which they are occurring, lead to an inability to engage with health‐care services. Disengagement from services and the absence of specialist care further isolates people, leaving them managing their diabetes alone with limited success. The development of severe complications is the unfortunate consequence and understanding this is an opportunity to consider service redesign. Listening to the views of people with T1DM about their clinic experiences allows HCP to learn of the unmet need for a more empathetic encounter that considers individual capacity and capability. Continuity in a tailored approach to care is vital to delay the onset of complications and would involve increased support for self‐management, monitoring for those who withdraw from care, and active endeavours to re‐engage with young people.

### 5.1 Limitations

Recruitment and data collection took place in a health‐care setting which influenced both the type of respondent and the recall of experiences thereby limiting the generalizability of findings to other community settings.

## CONFLICT OF INTEREST

None to declare.

## ETHICAL CONSIDERATIONS

The researchers obtained ethical approval to conduct this research from the Southern Adelaide Clinical Human Research Ethics Committee reference number 501.15.
